# Source Memory for Mental Imagery: Influences of the Stimuli’s Ease of Imagery

**DOI:** 10.1371/journal.pone.0143694

**Published:** 2015-11-25

**Authors:** Antonia Krefeld-Schwalb, Andrew W. Ellis, Margit E. Oswald

**Affiliations:** Institute of Psychology, University of Bern, Bern, Switzerland; The Ohio State University, Center for Cognitive and Brain Sciences, Center for Cognitive and Behavioral Brain Imaging, UNITED STATES

## Abstract

The present study investigated how ease of imagery influences source monitoring accuracy. Two experiments were conducted in order to examine how ease of imagery influences the probability of source confusions of perceived and imagined completions of natural symmetric shapes. The stimuli consisted of binary pictures of natural objects, namely symmetric pictures of birds, butterflies, insects, and leaves. The ease of imagery (indicating the similarity of the sources) and the discriminability (indicating the similarity of the items) of each stimulus were estimated in a pretest and included as predictors of the memory performance for these stimuli. It was found that confusion of the sources becomes more likely when the imagery process was relatively easy. However, if the different processes of source monitoring—item memory, source memory and guessing biases—are disentangled, both experiments support the assumption that the effect of decreased source memory for easily imagined stimuli is due to decision processes and misinformation at retrieval rather than encoding processes and memory retention. The data were modeled with a Bayesian hierarchical implementation of the one high threshold source monitoring model.

## Introduction

If one is asked to remember whether an event or object has been perceived or imagined, not only the object itself must be remembered, but also the encoding context, in this case the cognitive operation of perceiving or imagining the item, respectively. The encoding context of a memorized information is referred to as the source of a memory. It is stored or inferred from episodic memory. Both, source memory and item memory, need to interact in source monitoring, to come to a decision on the source of a memorized item. This process is influenced by the similarity of the items and the sources. Item memory accuracy decreases with similarity between items and distractors, and likewise, source memory accuracy decreases with similarity between possible sources [1–3].

This assumption can be transferred to the specific process of attributing sources to memories of visual mental imagery and visual perception. The sources are more likely confused if the memories of visual mental imagery and visual perception are similar. Features that heighten the similarity of memories of mental imagery to memories of visual perception are the vividness and the amount of details of the mental images, as well as a lack of records of the imagery process [4]. Several studies could show, that the ease of imagery, as well as the vividness of the mental images is associated with increased source memory confusions. For example, the experiments reported by Finke et al. [5] showed that source memory errors between memories of mental imagery and memories of visual perception are more likely for easily, compared to less easily imagined items. They interpreted the result as being caused by a lack of records of the imagery process, for easily imagined stimuli. Intraub and Hoffman [6] supported this account by showing that the awareness of the imagery process, was decisive for the confusion of sources. The awareness of the imagery process is dependent on the complexity and the difficulty of the imagery process. Furthermore, people with rather high imagery ability are more prone to confuse memories of mental imagery and visual perception, especially if imagery is not explicitly required [7, 8]. In general, confusions between mental imagery become more likely, if mental imagery was automatic or guided by external script, compared to deliberate imagery procedures [9]. However, mental imagery instructions change the response strategies at recognition, so that ease of mental imagery is used as a decision strategy at recognition [10]. If the item or only the source of the item are forgotten, subjects rely their response on the characteristics of the stimuli in such that more easily imagined items are more likely to be accepted as old. Because this response strategy does not rely on memorized information it is interpreted as biased guessing. Thus, decision strategies strongly affect the source attribution. Following this line of reasoning, differences in response probabilities, can sometimes equivalently be interpreted as being caused by differences in probabilities of item and source memory on one side or different guessing biases on the other side. Not only mental imagery influences the response strategy at recognition, but additionally, presentation of the stimulus in only one source in the recognition task might bias the source memory responses as well. When investigating the influence of mental imagery and the ease of mental imagery on source confusions, it must be considered that presenting only the lure item in the recognition task, as only the complete stimuli in the original work by Finke et al. [5], can bias the responses. Because, presenting the complete stimulus in the recognition task after the halved stimulus had been encoded can be understood as misinformation which lures the response “complete” [11] and stimulus specific ease of imagery might likewise influence this biasing effect.

The present study aims to answer the following question: Does ease of imagery enhance source similarity and following decrease the probability of source memory or does the ease of imagery rather bias the guessing tendencies at recognition? In order to answer that question we relied our experimental approach mainly on the experiments reported by Finke et al. [5]. The authors presented subjects with a series of halved and complete symmetrical shapes. The shapes were to equal parts, horizontal or vertical symmetrical. Before encoding the stimuli, subjects were instructed to memorize the following series of stimuli. It was manipulated between the subjects if they received imagery instructions, additionally to the memory instructions. Following the imagery instructions, the halved presented shapes had to be mentally completed to complete symmetrical shapes. Subsequently, at the recognition task, only the complete shapes were presented. Participants were asked whether the stimuli had been presented halved, complete or not at all during the encoding phase. Subjects more often falsely responded that the vertical symmetrical stimuli had been presented complete at encoding, than they falsely classified the halved presented horizontal symmetrical stimuli, only if they received imagery instructions. The authors concluded that ease of imagery leads to weaker source memory and the subsequent confusion of the sources, because the stimuli are more easily mentally completed to whole stimuli when presented as vertically symmetric, compared to when presented as horizontally symmetric. The authors interpreted this finding in line with the Source Monitoring Framework [12]. They inferred that the decrease in performance is caused by a lack of records of the mental imagery process and/or an increased amount of perceptual details in the memories of the easily generated images [5].

We followed this experimental design in most instances. However, in place of abstract shapes we used binary symmetrical images of natural objects. The application of natural objects heightens the external validity of the paradigm, compared to the application of abstract shapes. Additionally, instead of comparing groups of more and less easily imagined stimulus, we included the individual ease of imagery and discriminability of every stimulus as predictors. We expected the ease of imagery to decrease the probability of source memory and influence the corresponding guessing biases for the stimuli, whereas we expected the stimuli′s discriminability to increase the probability of item memory for the stimuli. Furthermore, additionally to the original study design, in which only the complete stimulus was presented at recognition, we conducted the experiment in two conditions. In one condition only the complete stimulus was presented at the recognition task and in the other condition both, the complete and halved stimulus were presented at the recognition task. We added this condition to the original design in order to test, whether the presentation of only the complete stimulus at recognition biases the responses and influences the effect of the stimulus features.

It is common sense in memory research that it is crucial to have measures of memory performance, which at least control for guessing probabilities, as contributing to the responses. Due to the fact that source memory performance is not only influenced by guessing probabilities, but also item memory, we must implement mathematical modeling techniques, which provide estimates of source memory, item memory and the guessing tendencies, based on the categorical responses in the source monitoring task. Threshold models of source memory, which belong to the multinomial processing tree MPT model class, provide a very well suited tool to estimate the mental states related to the decision process in a source monitoring task. The model parameters of MPT models of source monitoring, are interpreted to represent the probabilities of item memory, source memory, and the corresponding guessing biases [13, 14]. Most importantly, these models assume that the memory for the item, and for the source are either present or absent. If there is no memory, the memory status of the item is guessed with a certain bias and conditional on it the source of the item is guessed with another bias. These guessing biases can be influenced by the context, the task, and the features of the stimulus during the presentation of the stimuli at the recognition task. The models parameters were estimated with a hierarchical Bayesian approach, introduced by Klauer [15]. This approach enabled the consideration of heterogeneity between the stimuli and the prediction of the model parameters on the stimulus level, with the ease of imagery and the discriminability of the stimuli.

## Method

The data were collected in two experiments, which mainly differed with respect to the recognition task. The two experiments further differed in minor aspects, regarding the platform of the data collection, PsychoPy vs. unipark and the randomization of the stimuli. The allocation of trial conditions were randomized trial by trial in experiment one. Contrarily, randomized lists of trials were applied in Experiment 2. Importantly, these differences did not change the visual appearance of the stimulus presentation at the encoding phase.

### Participants

A total of 234 undergraduate psychology students participated in the experiments for course credits. The study was approved by the ethical committee of the University of Bern. All participants gave written informed consent prior to data collection. No personal data was collected. The data can not be assigned to the individual participants.

### Materials

**Fig 1:**
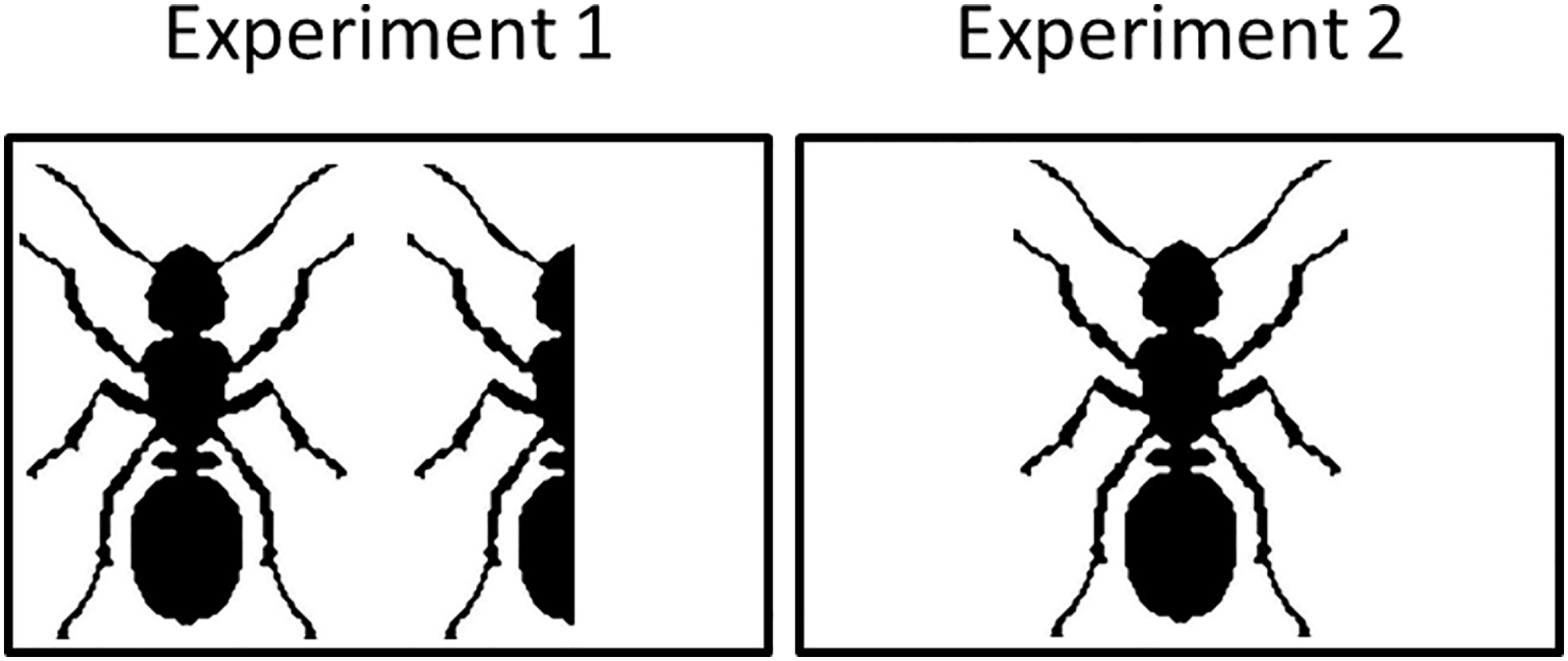
Stimulus presentation in the recognition tasks of Experiment 1 and Experiment 2. Halved and complete presentation in Experiment 1 and complete presentation only in Experiment 2.

A set of stimuli (N = 80) were created based on binary pictures of natural objects from the LEMS Database [16]. Binary pictures means that all pixels in these pictures are either black or white, grayscales are depicted as black. As the stimuli needed to be symmetrical and without perceptual preference for either axis of symmetry, binary pictures of birds, butterflies, leaves and insects, which are naturally symmetrical, were chosen. The stimuli thereafter consisted of 40 stimuli in each of the vertical and horizontal symmetric orientations. One of the stimuli is presented as vertically symmetrical in Fig 1. Each stimulus was represented twice in the stimulus pool. To optimize symmetry, the binary pictures were cut and mirrored on the symmetrical axes. The ease of imagery of the stimuli was rated in a pretest with 54 subjects. Importantly, the subjects rated horizontal and vertical symmetrical stimuli, but never rated two versions of the same stimulus. They either rated the vertical or the horizontal symmetrical stimulus. The ease of imagery (EI) was derived in a principal component analysis of multiple measurements: the rated ease of imagery, the duration of the imagery process indicated with key pressing, the rated complexity of the stimulus, the rated familiarity of the stimulus and the relative number of trials in which subjects recognized the same object in the stimulus. Subjects had to decide, if they recognized anything in the stimulus, and if so to specify, what they recognized. Subsequently, after the rating process in the pretest, the recognition of the stimuli was tested. As a measure of the discriminability of one stimulus from the other stimuli′s, the d′s [17] were calculated for each stimulus. For further details on the pretest, as well as a depiction of the horizontal symmetrical versions of the stimuli, see S1 File and S1 Fig. The stimuli were presented in a white rectangle; the visual angle was approximately 14 degree.

### Design

The experiment was conducted under 2×2 conditions between the subjects: with or without imagery instructions (No-imagery vs. Imagery) and either under presentation of only the complete stimulus, or the complete and the halved stimulus at the recognition task (complete vs. both). The latter factor was manipulated between the subjects in two experiments, which had been conducted in separate data collection procedures. A total of 148 participants participated at Experiment 1 under presentation of both stimulus versions at the recognition task, whereas 86 participants conducted Experiment 2 under the presentation of only the complete version of the stimuli at the recognition task, see Fig 1. The presentation of the stimuli was randomized trial by trial in Experiment 1. Following, not every stimulus appeared an equal number of times under the conditions. It was controlled that every subject either saw the horizontal or the vertical symmetrical version of a stimulus, not both versions. In Experiment 2, lists of trials have been constructed beforehand, to balance that the stimuli were presented an equal number of times in the different conditions.

### Procedure

The participants were advised that they would be participating in an experiment on memory and visual perception. All subjects knew that the encoding phase would be followed by a recognition test, however no instruction about the source test was given. Under the imagery condition, the completion of the halved presented stimuli was additionally illustrated by an exemplar stimulus. Subjects were alternately assigned to the imagery, or no-imagery, condition. Under all conditions, subjects were exposed to 20 of the 80 stimuli. The order, the presentation (halved or complete) and the symmetric orientation of the stimuli were randomized in Experiment 1 and randomized and balanced in Experiment 2. Each stimulus presentation lasted 7000ms and was preceded by a fixation cross for 1000ms. Every stimulus was presented in a white rectangle; the visual angle was approximately 14, in the center of the desktop on a gray background screen. If the stimulus was presented complete, the subjects were instructed to memorize the stimulus; the instruction appeared simultaneously with the stimulus and lasted for 1000ms. Under the no-imagery condition both halved and complete presented stimuli had the same instructions. Whereas, the subjects in the imagery condition were instructed to mentally complete the stimuli to form symmetric stimuli. When the stimuli were presented as halved, the left side of the stimulus was always shown when vertically symmetrical. The upper side of the stimulus was always shown when presented horizontally symmetrical. The axis of symmetry always crossed the center of the screen. After the 20 stimuli had been presented, the subjects conducted a short distractor task: the need for cognition questionnaire [18]. Subsequently, the recognition task started. Every stimulus which had been encoded, as well as 20 distractor stimuli were tested in the recognition task. In Experiment 1, all the stimuli were presented in both versions, halved and complete adjacent to each other. The halved stimulus always appeared on the left, and the complete stimulus always appeared on the right side of the center—again in two white rectangles of 400×400 pixels with the symmetric axes crossing the centers of these white rectangles. With the aid of these stimuli, participants were requested to decide how the stimulus had been presented at the encoding phase (“halved”, “complete” or “not at all”). The subjects had to click on the particular check boxes under the stimuli and confirm their choice by another check box. There was no time restriction on the trials in this task. The procedure of the recognition task of Experiment 2 was the same, besides that only the complete stimulus was presented. After the subjects had given their responses for each of the 40 stimuli, they were debriefed. Subjects received no feedback on their performance in the task.

### Data Analysis

The Bayesian hierarchical MPT model, which was implemented in the present study, has been introduced in the latent-trait approach by Klauer [15]. The model parameters were estimated for every stimulus. Given the data, which provide 6 free response categories, the complete MPT model of source monitoring has more parameters than degrees of freedom, see Table 1. Following, the model is not identified, when all parameters are free to vary and some parameters must be restricted to reach identifiability. The parameters and restrictions, of the implemented model are depicted in Table 1. The probability of the correct rejection of the distractor items was restricted to be zero, *D*3 = 0. This restriction represents a one-high-threshold model, compared to a two-high-threshold model with *D*3 > 0. Implementation of the two high threshold model, resumed in unreasonably inflated source memory parameters [19]. The parameters of the hierarchical MPT model and its fit were estimated, via Bayesian modeling techniques. In the hierarchical Bayesian model, as proposed by Klauer [15] it is assumed, that the individual parameters *θ*_*sp*_ are given by the sum of a parameter specific mean *μ*_*p*_ and the individual deviance from this mean *δ*_*sp*_. For the purpose of the present study, this summation was extended with parameter specific predictors of the model parameters *β*1 and *β*2. These predictors represent the influence of the discriminability d’ and the ease of imagery EI of the stimuli. These variables were derived from the rating procedure and the recognition test in the Pretest. Thus, the model parameters *θ* are given by:

$$\begin{array}{c} \theta_{sp}=\mu_{p}+\beta 1*d_{s}^{\prime }+\beta 2*EI_{s}+\delta_{sp} \end{array}$$ (1)

**Table 1:** Parameters and their restrictions in the implemented MPT model.

Parameter	Interpretation
*D*1	Probability of item memory for complete presented stimuli
*D*2	Probability of item memory for halved presented stimuli
*D*3 = 0	Probability of correct rejection of distractor items
*d*1	Probability of source memory for complete presented stimuli
*d*2	Probability of source memory for halved presented stimuli
*a*	Probability to guess that the item has been presented halved, without source memory
*b*	Probability to guess that the item has been presented
*g* = *a*	Probability to guess that the item has been presented halved, without source and item memory

The priors of *μ*_*p*_, *β*1_*p*_ and *β*2_*p*_ for every p were independent normal distributions with *μ* = 0 and *σ* = 1 [20]. These distributions correspond to uniform distributions on the probability scale. However, the prior for the item effect *δ* was a multivariate distribution with *μ*_*p*_ = 0_*P*_, and Σ. The hyperprior of the covariance matrix Σ was a scaled inverse-Wishart distribution [21]. The sampling software JAGS was used to augment the data, and infer the posterior distributions of the parameters. The data (S1, S2, S3, and S4 Data), both models (S3 and S4 Files), as well as the code for the Bayesian inference via R and JAGS (S2 File), can be found in the supplementary material of this article.

## Results

The mean relative response frequencies are depicted in Table 2. Inspecting these behavioral results, performance was comparable between the conditions, however slightly worse in Experiment 2, under the presentation of only the complete stimulus at recognition, compared to Experiment 1 and in the conditions with imagery instructions, compared to the conditions without imagery instructions. The experiments and conditions will be compared in detail in terms of the estimated model parameters.

**Table 2:** Relative response frequencies of the response categories in the experimental conditions.

Presentation	Complete			Halved			New		
Response	*complete*	*halved*	*new*	*complete*	*halved*	*new*	*complete*	*halved*	*new*
Exp1Imag	.60	.24	.16	.13	.67	.20	.11	.22	.67
Exp1NoImag	.67	.19	.14	.11	.69	.0	.13	.20	.67
Exp2Imag	.52	.24	.24	.19	.48	.33	.09	.24	.67
Exp2NoImag	.70	.16	.14	.12	.53	.35	.11	.21	.68

### Model Fit

The hierarchical MPT model was separately fitted to the conditions. The deviance information criteria DIC as well as the indicators of the posterior predictive model checks are depicted in Table 3. The DIC indicated similar fit of the data to the model in the different conditions. Likewise, the posterior predictive checks indicated good predictions of the model, in all conditions, as *p* is neither close to 0 nor close to 1 [21]. As a test statistics for the posterior predictive model checks, the sum and squared residuals of the data T1 and the predicted data T2 were subtracted.

**Table 3:** Model fit statistics.

	DIC	*p*	T1	T2
Exp1Imag	1494.39	0.48	1435.34	1426.20
Exp1NoImag	1487.70	0.62	1275.87	1321.58
Exp2Imag	1351.72	0.36	1043.44	1007.04
Exp2NoImag	1365.338	0.31	982.83	935.00

*Note*. *p* = indicator of the relative amount of the amount of residuals in the data T1 and predicted data T2

### Influence of stimulus presentation in the recognition task

The means as well as the 95% highest posterior density intervals 95% HDI of the parameters in the different conditions are depicted in Table 4. As can already be seen in this depiction, the parameters differed remarkably between the conditions. To report the differences in the parameter estimates we will report the mean differences as well as the *p*-values which represent the relative number of samplings from the posterior distribution of the mean differences, which are greater than 0. Most importantly, the item memory estimates *μ*_*Diff*: *D*2_ = 1.01, *p* > .99, as well as the source memory *μ*_*Diff*: *d*2_ = 1.51, *p* = .98, for halved presented Items were lower in Experiment 2, under the presentation of only the complete stimulus at recognition, in the imagery condition. Likewise the item memory for complete presented items was lower in Experiment 2, *μ*_*Diff*: *D*1_ = 0.61, *p* > .99. There was no difference in the guessing biases for the source *μ*_*Diff*: *a*_ = −.151, *p* = .873. However, there was a noteworthy difference in the guessing biases for the item memory *μ*_*Diff*: *g*_ = − .462, *p* < .001. Thus, under the presentation of only the complete stimulus at the recognition task, subjects were more likely to accept the stimulus as old. Furthermore, subject had less accurate memory in this experiment. The differences under the no-imagery condition were very similar and differed only with regard to the size of the effect from the effects under the imagery Condition.

**Table 4:** Means and 95% HDI of the model parameters in the different conditions.

	Exp1Imag		Exp1NoImag		Exp2Imag		Exp2NoImag	
	*μ*	95%*HDI*	*μ*	95%*HDI*	*μ*	95%*HDI*	*μ*	95%*HDI*
*μ*_*D*1_	0.76	[0.71, 0.81]	0.80	[0.75, 0.85]	0.53	[0.41, 0.64]	0.71	[0.61, 0.80]
*μ*_*D*2_	0.71	[0.65, 0.76]	0.71	[0.65, 0.76]	0.32	[0.19, 0.46]	0.42	[0.29, 0.55]
*μ*_*d*1_	0.67	[0.59, 0.74]	0.74	[0.66, 0.81]	0.67	[0.46, 0.85]	0.78	[0.61, 0.92]
*μ*_*d*2_	0.61	[0.39, 0.80]	0.78	[0.63, 0.89]	0.11	[0.00, 0.53]	0.42	[0.07, 0.84]
*μ*_*a*_	0.67	[0.62, 0.71]	0.65	[0.59, 0.70]	0.72	[0.64, 0.79]	0.73	[0.63, 0.81]
*μ*_*b*_	0.31	[0.27, 0.36]	0.31	[0.28, 0.35]	0.49	[0.41, 0.56]	0.46	[0.39, 0.54]

### Influence of the stimuli’s discriminability d’ and ease of imagery EI

The regression weights, predicting these parameters with the stimuli’s d’ and EI are depicted in Tables 5 and 6, respectively. The logarithmic Bayes Factors log(BF) were calculated with the Savage-Dickey density ration [22]. Positive log(BF) indicate results in favor of the H1 and negative log(BF) indicate results in favor of the H0. Logarithmic BF greater than *log*(*BF*) > 1.1 provide evidence worth mentioning, for the H1. Logarithmic BF smaller than *log*(*BF*) < −1.1 provide evidence worth mentioning, for the H0.

**Table 5:** Means and logarithmic Bayes Factors in parenthesis of the regression weight *β*1, indicating the influence of the stimuli’s discriminability d’.

	Exp1Imag			Exp1NoImag			Exp2Imag			Exp2NoImag		
	*μ*	95%*HDI*	log(BF)	*μ*	95%*HDI*	log(BF)	*μ*	95%*HDI*	log(BF)	*μ*	95%*HDI*	log(BF)
*β*1_*D*1_	0.15	[0.00, 0.31]	−0.54	0.28	[0.13, 0.44]	3.26	0.70	[0.24, 1.20]	3.14	0.54	[0.07, 1.01]	1.11
*β*1_*D*2_	0.23	[0.08, 0.38]	2.13	0.15	[−0.01, 0.32]	−0.77	0.94	[0.39, 1.55]	5.88	0.35	[−0.16, 0.89]	−0.46
*β*1_*d*1_	0.07	[−0.13, 0.27]	−2.03	0.06	[−0.17, 0.27]	−2.07	0.22	[−0.54, 1.03]	−0.80	0.46	[−0.41, 1.28]	−0.24
*β*1_*d*2_	0.27	[−0.24, 0.85]	−0.87	0.36	[−0.07, 0.87]	−0.20	−0.71	[−2.35, 0.87]	0.13	0.09	[−1.54, 1.66]	−0.22
*β*1_*a*_	−0.02	[−0.14, 0.09]	−2.75	−0.01	[−0.16, 0.14]	−2.59	0.33	[−0.07, 0.74]	−0.21	0.12	[−0.43, 0.66]	−1.19
*β*1_*b*_	−0.28	[−0.40, −0.15]	5.57	−0.30	[−0.42, −0.19]	7.41	−1.06	[−1.41, −0.79]	12.64	−0.96	[−1.33, −0.59]	8.08

**Table 6:** Means and logarithmic Bayes Factors in parenthesis of the regression weights *β*2, indicating the influence of the stimuli’s ease of imagery EI.

	Exp1Imag			Exp1NoImag			Exp2Imag			Exp2NoImag		
	*μ*	95%*HDI*	log(BF)	*μ*	95%*HDI*	log(BF)	*μ*	95%*HDI*	log(BF)	*μ*	95%*HDI*	log(BF)
*β*2_*D*1_	0.12	[−0.06, 0.28]	−1.45	−0.04	[−0.20, 0.12]	−2.4	−0.16	[−0.34, 0.03]	−1.03	−0.05	[−0.23, 0.12]	−2.22
*β*2_*D*2_	0.06	[−0.09, 0.22]	−2.27	0.06	[−0.10, 0.22]	−2.27	0.21	[0.00, 0.41]	−0.23	0.01	[−0.19, 0.2]	−2.34
*β*2_*d*1_	−0.14	[−0.39, 0.08]	−1.47	0.01	[−0.22, 0.24]	−2.16	0.15	[−0.22, 0.57]	−1.41	−0.22	[−0.57, 0.09]	−0.85
*β*2_*d*2_	0.06	[−0.42, 0.57]	−1.41	−0.28	[−0.74, 0.12]	−0.7	−0.18	[−1.23, 0.78]	−0.68	−0.77	[−1.97, 0.29]	0.52
*β*2_*a*_	−0.17	[−0.28, −0.06]	1.84	−0.06	[−0.18, 0.07]	−2.33	−0.27	[−0.42, −0.12]	2.89	−0.18	[−0.35, −0.01]	−0.29
*β*2_*b*_	0.20	[0.07, 0.32]	1.30	0.21	[0.10, 0.32]	3.31	0.23	[0.12, 0.34]	4.5	0.23	[0.11, 0.35]	3.78

*D1*, The item memory parameter for complete presented items, were in all conditions predicted by the stimulus d’, except for Experiment 1 in the imagery condition. In the imagery condition, *D2*, the probability of item memory of halved presented items, was even so predicted by the stimuli’s d’. The stimulus’ d’ increased the probability to correctly remember the stimuli. Consistently over all conditions, *b*, the probability to guess that the stimulus had been presented, was decreased with the stimuli’s d’. This negative effect is explained by the nature of d’ which decreases with the probability of false positives. There is no evidence for or against an effect of the stimuli’s d’ on *d2*, the probability of source memory for halved presented items in the imagery condition and in both conditions of Experiment 2. There was only evidence in the no-imagery condition of Experiment 1 that, *d1* source memory for complete presented items, was not predicted by the stimuli’s d’. Thus, the stimuli’s discriminability heightens the item memory of the stimuli, but it is also negatively related to the tendency to accept the stimulus as old, if the stimulus is actually forgotten.

In the imagery condition of Experiment 1, *d2*, source memory for halved presented stimuli, was not predicted by the EI of the stimuli. However, there is no evidence for either an effect or no effect of EI on *d2* in the other conditions. In both conditions of Experiment 1 and in the imagery condition of Experiment 2, *d1*, the probability of source memory for complete presented stimuli, was not predicted by the EI of the stimuli. In the imagery condition of Experiment 1, *d2*, source memory for halved presented stimuli, was not predicted by the EI of the stimuli, as well.

Likewise, *D1* and *D2*, the probabilities of item memory, were not predicted by the stimuli’s EI, in Experiment 1. Only in the imagery condition in Experiment 2, there was not enough evidence against the effect. Contrarily, the stimulus’ EI increased *b*, the probability to guess that the stimulus had been presented, consistently over all conditions. Under the imagery conditions, it additionally predicted *a*, the probability that the stimulus is guessed to have been presented complete. However, there is no effect on *a* in the no-imagery condition of Experiment 1. To conclude, memory accuracy is not predicted by the stimuli’s EI. However, the guessing tendencies for the source and the item are related to the stimuli’s EI.

## Discussion

The present study gives additional support for the influence of the ease of mental imagery on the confusion of sources of memories of mental imagery and visual perception. Evidence can be provided, that the probability of false source attribution for a given stimulus increases with the ease of imagery of this stimulus. However, this effect is driven by biased response strategies at recognition, instead of decreased source memory for easily imagined stimuli. We cannot provide evidence for a decreasing effect of the ease of imagery of the stimuli on the probability of source memory. Furthermore, the presentation of only the lure stimulus at recognition substantially decreases the probabilities of item memory for halved presented items and changes the guessing tendencies.

So far, the findings are in line with the findings of Finke et al. [5] and others, for a review see Henkel and Carbuto [23]. But our findings cannot support their interpretation of decreased probability of source memory at all costs. It is one of the core ideas of the source monitoring framework [12] that the similarity of the sources decreases the accuracy of source monitoring, or rather, that it leads to the confusion of the sources. Applied to source monitoring of memories of mental imagery and visual perception, the ease of imagery is hypothesized to heighten the similarity of the sources of theses memories. The similarity is increased on mainly two dimensions. First, ease of imagery decreases the probability of records of the imagery process. Second, ease of imagery increases the amount of perceptual details in the mentally created images [5]. We could replicate the finding that sources are more likely confused if the sources were relatively similar. But, this decrease of source monitoring accuracy is not due to a decrease of the probability of source memory. If item memory, source memory and guessing biases are disentangled via modeling procedures, the similarity of the sources does not influence the probability of source memory. Rather, the guessing bias for one source and the guessing bias for the presence of the item are influenced by the stimulus specific ease of imagery. This effect indicates that under the imagery conditions subjects use mental imagery at recognition, and the ease of the imagery process, as a cue of the memory status of the item, if the item was forgotten and as a cue for the source of the item, if the source is forgotten, too.

However, the probability of item memory is predicted by the discriminability of the stimuli. Following, the similarity of the items, which is indicated by their discriminability, does influence the accuracy of the memory for the items. Performance in the imagery conditions was generally decreased compared to the no-imagery conditions. This finding contrasts with the generating effect [24]. But, the memory decreasing effect of imagery instructions has been observed in several source monitoring experiments [7, 8]. Finke et al. [5] suggested that whether the effect of imagery instructions is positive of negative depends on the ratio of costs and benefits of the generated images. Following, the imagery process, in the present experiments, could not successfully be used as a cue for the memory. But rather, mental imagery decreased the probability of item and source memory. This finding can be explained by an overall ease of the imagery process in the present experiments. Imagery might have been mostly effortless, and even automatic, so that no additional cognitive operation was recorded and could be used as a cue or the source at recognition, see also [9]. Following, the generated images produced more costs than benefits for the accuracy of the memory. Additionally, presentation of the lure in the recognition task further decreased memory, especially under the conditions with imagery instructions. Assuming that subjects must rely their response on guessing, based on the stimulus characteristics, if the item and or the source is forgotten, it is to be concluded that mental imagery is used as response strategy more often in these conditions [10]. Because the ease of mental imagery influences the guessing tendencies for the source and the item. However, ease of mental imagery as a stimulus specific feature does not only influence the mental imagery process, but also encoding processes in the recognition task. Especially symmetry, which was used to manipulate the ease of mental imagery in the original design by Finke et al. [5], does not only manipulate the ease of imagery, but also the ease of general visual processing and encoding fluency [25, 26]. More fluently perceived shapes increase the tendency to accept these shapes as old [27]. In order two prove that the effect of ease of mental imagery does no only influence decision processes, but actually enhances the similarity of memories of mental imagery and visual perception, as stated by the Source Monitoring Framework [12], it would be necessary to manipulated ease of imagery as a variable which does only influence the imagery process and not encoding processes in the recognition task.

Altogether our findings give additional support for the idea, that the effect of decreased accuracy of source monitoring for easily imagined stimuli is driven by decision processes and misinformation effects at recognition, rather than processes during encoding and memory retention. If the item and the source, or only the source, are forgotten subjects rely their guessing on the features of the stimuli, the discriminability of this stimulus to the other stimuli, and the stimulus specific ease of imagery. The findings are supported by two experiments with different recognition tasks and data analysis via hierarchical Bayesian modeling techniques.

The data was modeled with a threshold model of source memory. Instead of a threshold model, a continuous model such as a multivariate hierarchical signal detection model [28] could also have been implemented to model the data. It is an ongoing debate which of these model classes are most suited to model source memory [2, 29–35]. However, reviewing the complete debate and contributing to it, is beyond the scope of this article. Nonetheless, I will point out the main reasons, for which we decided us for the application of a multinomial processing tree model. First, both models make very similar predictions [29, 36, 37]. Second, MPT models provide a very useful and intriguing measurement tool of source memory. Third, the main critics on MPT models could be extenuated [30, 38, 39]. Although the debate has not been resolved yet, we rely on the MPT as well suited measurement tool for the given data.

The model selection procedure within different MPT models given the present data revealed that the data of both experiments are best described with 1HTSMs, although the 1HTSM has been criticized [40, 41] of not being able to account for source memory and recognition data. Other authors still rely on the 1HSTM as a valid measure of source memory and recognition memory [42]. Menon And Woodward [19] even provide evidence in favor of the 1HTSM over the 2HTSM in a direct comparison, based on simulated data. The models provided a good fit to the data, however there was considerably high uncertainty in the estimation of the source memory parameters in Experiment 2. This variance might be reduced by additionally controlling for between subjects variability. Despite this, the negative Bayes Factors show that there is considerable evidence in favor of the zero hypotheses in most of the cases, when the hypotheses in question were not supported.

In order to extent our findings, some extensions of our experimental designs, could be made. For example, a greater number of items would increase the certainty in the estimates. The obvious advantage of the stimuli—that they are only slightly edited binary pictures of natural objects, and thereafter heightening the validity of the paradigm—was, at the same time, their disadvantage. The stimulus pool was restricted and could not be easily enlarged. Following, the number of trials per participant was restricted and the ease of imagery was extensively rated, but could not be directly manipulated. The same is true for the discriminability of the items. The measurement of the d’, as well as the ease of imagery, could be improved, and the measurement error could be included in the model. A more elegant approach would enable direct, stepwise manipulations of the ease of imagery and the discriminability of the items. Or, more generally spoken: two independent manipulations of the similarity of the sources and the similarity of the items. There is one last constraint to be mentioned that refers to the task used in this experiment. Imagery was not inherent in the task, but had to be demanded via the instructions. It is not guaranteed, and could not be controlled, that the subjects were actually imagining the stimulus in the imagery condition and were not imagining it in the no-imagery condition. The imagery process was not necessary to solve the task correctly or to solve it in a more effective manner. Nevertheless, the presentations can under both conditions be regarded as different sources, as the stimuli were presented halved or complete at encoding under the both conditions.

To sum up, the present study is, besides its limitations, a new and important approach to investigate source monitoring of memories of mental imagery and visual perception more closely. Our results stress the assumption of increased source similarity and decreased source memory for easily imagined items and support the idea of the effect of mental imagery, lure presentation and the ease of imagery on the response strategies in a source monitoring task.

## Supporting Information

S1 Fig Depiction of the stimuli. (PDF)

S1 File Summary of the results of the Pretest, the validation of the stimulus material. (PDF)

S2 File R code for running the models in JAGS. (R)

S3 File JAGS code of the 1 HTSM modell. (R)

S4 File JAGS code of the 2 HTSM modell. (R)

S1 Data Data of the imagery condition of Experiment 1. (CSV)

S2 Data Data of the no-imagery condition of Experiment 1. (CSV)

S3 Data Data of the imagery condition of Experiment 2. (CSV)

S4 Data Data of the no-imagery condition of Experiment 2. (CSV)
