# Supplementary figures and images for: Source Memory for Mental Imagery: Influences of the Stimuli’s Ease of Imagery
Source: PLoS One. 2015 Nov 25;10(11):e0143694. doi: 10.1371/journal.pone.0143694 (PMC4659550; doi:10.1371/journal.pone.0143694)

Leaves

Birds

Butterflies

Insects

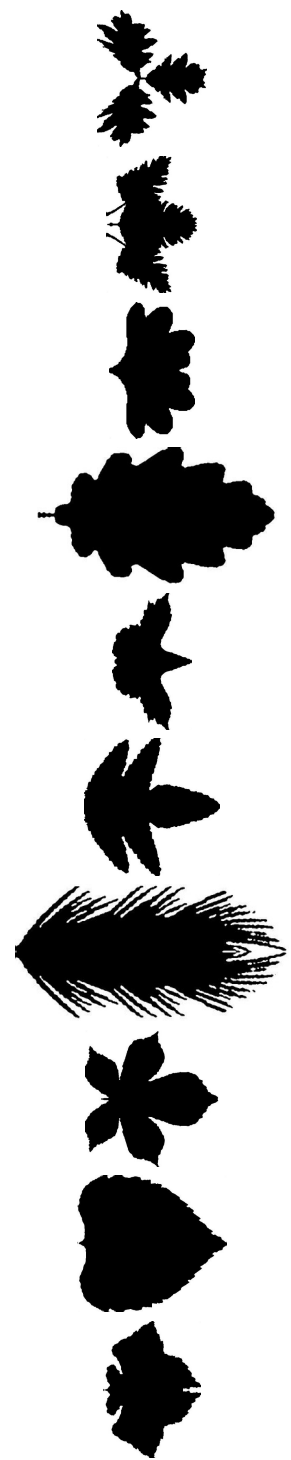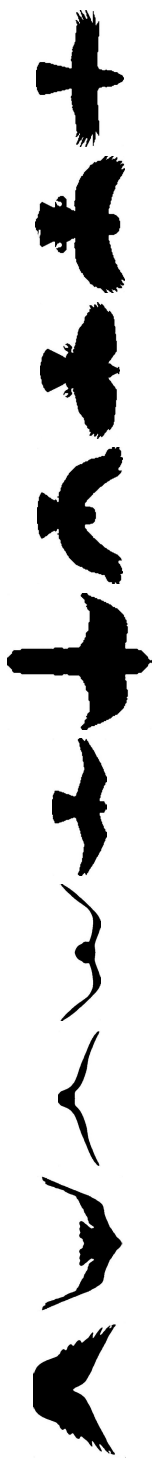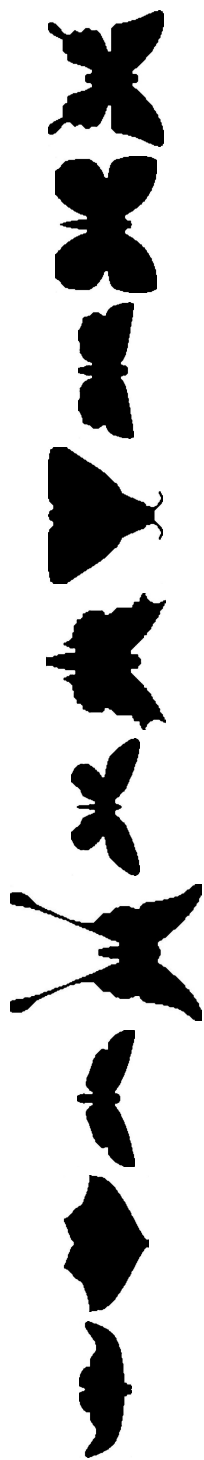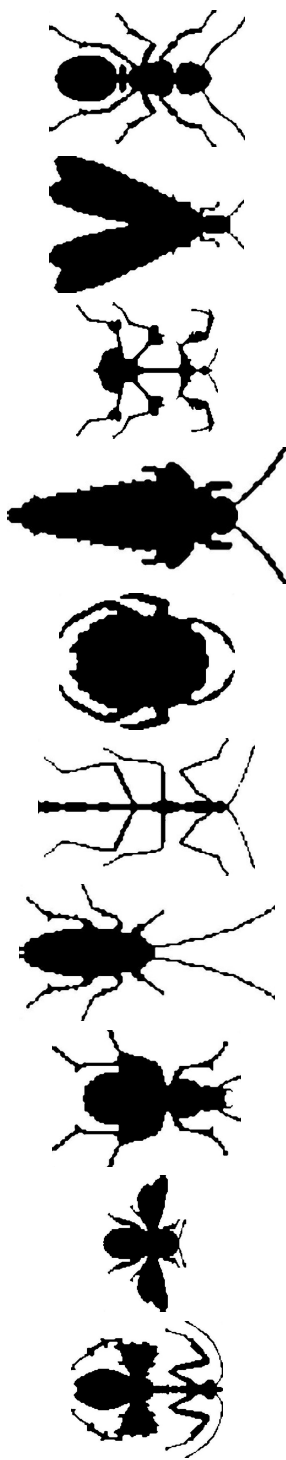

Supplement: S1 Fig — (PDF) [file pone.0143694.s001.pdf]
