# Supplementary material for: Source Memory for Mental Imagery: Influences of the Stimuli’s Ease of Imagery
Source: PLoS One. 2015 Nov 25;10(11):e0143694. doi: 10.1371/journal.pone.0143694 (PMC4659550; doi:10.1371/journal.pone.0143694)
Supplement: S1 File — (PDF) [file pone.0143694.s002.pdf]

Pretest: Stimulus Validation

Encoding Presentation of 20 halved stimuli, completion of the stimuli to symmetrical stimuli was demanded and subjects had to rate each stimulus on 4 variables, while the presentation of the stimulus:

Recognition Recognition test for the, now completely presented 20 stimuli and 20 distractor stimuli. The results of the recognition test were used to calculate the discriminability of the items *d*' for every stimulus.

Results The ratings of the stimulus features were strongly inter-correlated, but not significantly correlated with the discriminabilitiy of the stimuli, *d*'. A principle component analysis of stimulus features was conducted, resuming in s single factor structure with the factor ease of imagery (EI). EI explains 79.33% of the variance and has an eigenvalue of 3.97. Intercorrelation of stimulus features, and discriminability *d*' and correlation with extracted factor from PCA are depicted in the following Table.

Table 1

Inter-correlations of stimulus features, and discriminability d' and correlation with extracted factor from PCA

Pearson’s r (n=80)	dImag	eImag	com	fam	sem	EI	d’
dImag	-	-.79***	.59***	-.75***	-.70***	-.86***	-.03
eImag		-	.82***	.89***	.78***	.96***	-.02
com			-	-.65***	-.57***	-.83***	.09
fam				-	.86***	.94***	-.04
sem					-	.86***	.09
